# Duodenal diversion surgery in management of intractable tracheobroncho-gastric fistula after esophagectomy

**DOI:** 10.1186/s44215-023-00072-z

**Published:** 2023-09-20

**Authors:** Masayuki Urabe, Koichi Yagi, Shuntaro Yoshimura, Motonari Ri, Shoh Yajima, Yasuhiro Okumura, Yasuyuki Seto

**Affiliations:** Department of Gastrointestinal Surgery, Graduate School of Medicine, the University of Tokyo, 7-3-1 Hongo, Bunkyo-ku, Tokyo, 113-8655 Japan

**Keywords:** Duodenal diversion, Esophagectomy, Tracheobronchial fistula

## Abstract

**Background:**

Tracheobroncho-gastric fistula (TGF), a rare but potentially fatal complication following esophagectomy with gastric conduit reconstruction, has conventionally been treated with surgical repair and/or airway stenting. However, satisfactory therapeutic outcomes with these modalities have yet to be obtained because of difficulty in controlling persistent inflammation caused by digestive juice reflux.

**Case presentation:**

We adopted duodenal diversion (DD), a classic anti-reflux surgical method, as an additional option for TGF management and have experienced two cases undergoing DD surgery for post-esophagectomy TGF (all male, 76–77 years old). TGF was developed after gastric conduit necrosis and anastomotic leakage, respectively, in these patients. In both cases, the DD procedure combined with surgical fistula repair was feasible with no DD-related complications. These operations achieved a good effect in terms of preventing gastroduodenal reflux and ameliorating respiratory status. Reconstructive surgery after DD was performed and oral dietary intake was successfully resumed in one case.

**Conclusion:**

DD appears to be a valid evacuation therapy when airway contamination with gastroduodenal reflux is not amenable to the conventional approach alone, and can usefully be included in the TGF treatment strategy in appropriate cases.

## Background

Esophageal cancer (EC) is an aggressive disease with an unfavorable prognosis. Esophagectomy with regional nodal clearance has been accepted as the mainstay for EC treatment, but is strongly associated with postoperative morbidity and even mortality [1]. Fistula formation between aerodigestive organs, while rare, is a potentially life-threatening event associated with EC surgery [2]. Tracheobroncho-gastric fistula (TGF) developing after esophagectomy with gastric conduit reconstruction can trigger critical pulmonary inflammation, and the associated mortality rate is reportedly as high as 30–42% [2–4]. Conventional treatment strategies for TGF include surgical procedures such as drainage, suturing closure and vital tissue flap transposition [4–7], as well as non-surgical procedures represented by tracheal stent implantation [8–10]. According to recent comprehensive studies, outcomes following these treatments are still unsatisfactory with 9-month survival rates of only 13–38% [11, 12]. These results reflect the difficulty in controlling severe respiratory contamination induced by digestive juice influx through the TGF.

Duodenal diversion (DD) has classically been recognized as an effective surgical method for preventing duodenogastric reflux [13, 14]. Furthermore, a previous prospective randomized trial actually demonstrated that gastric reconstruction with DD plus Roux-en-Y anastomosis yielded significantly better quality of life, as regards reflux-related symptoms after esophagectomy, than standard gastric reconstruction [15]. Given these background factors, we adopted DD surgery as an evacuation treatment option for post-esophagectomy TGF when respiratory contamination was persistent and not controllable with conventional therapeutic modalities alone. We retrospectively reviewed our experiences with DD operations and discuss herein the therapeutic relevance in managing intractable TGF.

## Case presentation

Between September 2016 and August 2022, a total of 296 consecutive patients underwent subtotal esophagectomy with gastric conduit reconstruction for EC in our institution. Postoperative TGF developed in two of these cases (0.7%), both of whom eventually underwent DD surgery. We retrospectively reviewed the clinicopathological characteristics and therapeutic courses of these cases. Clinical staging of tumors was based on the TNM classification (UICC, eighth edition). This analysis was carried after obtaining approval from the Ethics Committee of our institution (no. 3962). Informed consent was obtained from the two subjects for being included in this study.

The clinical presentations of our cases are described below. In both patients, the bronchial arteries were carefully preserved during esophagectomy and there were no injuries of the tracheobronchial tree. Postoperative TGF was diagnosed based on esophagogastroduodenoscopy (EGD) and fiberoptic bronchoscopy findings. Basic surgical procedures for DD were as follows. First, the branches of the supra- and retroduodenal vessels supplying the duodenum were partially ligated, with the gastroepiploic and right gastric vessels being completely preserved. Second, the duodenum was separated distal to the pyloric ring using a linear stapler. A decompression gastrostomy tube was finally inserted into the duodenal stump of the gastric conduit side. Unless already present, a nutritional jejunostomy access was constructed.

### Case 1

**Fig. 1:**
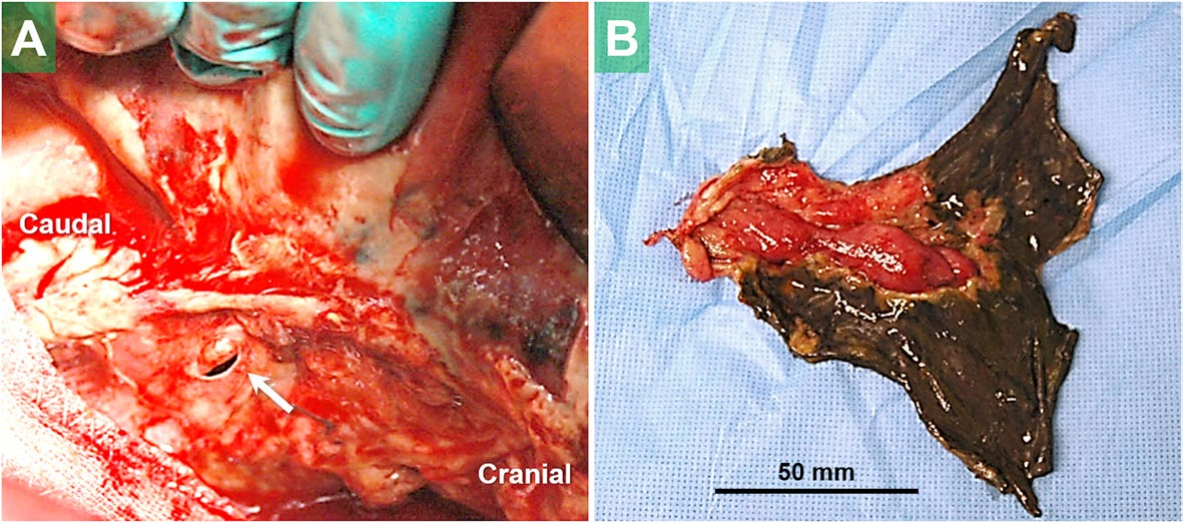
A membranous hole of the right bronchus (*arrow*) was intraoperatively detected (**A**). The necrotic portion of the gastric conduit was resected (**B**)

A 76-year-old man, receiving maintenance hemodialysis for diabetic renal failure, underwent upfront radical surgery for cT3N1M0 EC (squamous cell carcinoma). The operation was comprised of a right-thoracotomy esophagectomy, two-field lymphadenectomy, mediastinal gastric conduit reconstruction and tube jejunostomy. On the 4th postoperative day (POD), postcontrast computed tomography (CT), conducted as part of diagnostic work-up for fever, revealed extensive gastric conduit ischemia. On the 5th POD, gastric conduit necrosis was confirmed endoscopically and concurrent respiratory dysfunction necessitated tracheal intubation and mechanical ventilation. On the 6th POD, surgical drainage was performed via the cervical incision. On the 12th POD, bronchoscopy identified a bronchial membrane fistula connected to the gastric conduit. We thus performed an emergent right-thoracotomy consisting of (i) resecting the necrotic portion of the stomach, (ii) patching the bronchial defect with an intact portion of the gastric wall and (iii) creating a cervical esophagostomy (Fig. 1). In order to save time, we opted to use the well-supplied part of the stomach as a vital tissue patch rather than the muscular flap.

**Fig. 2:**
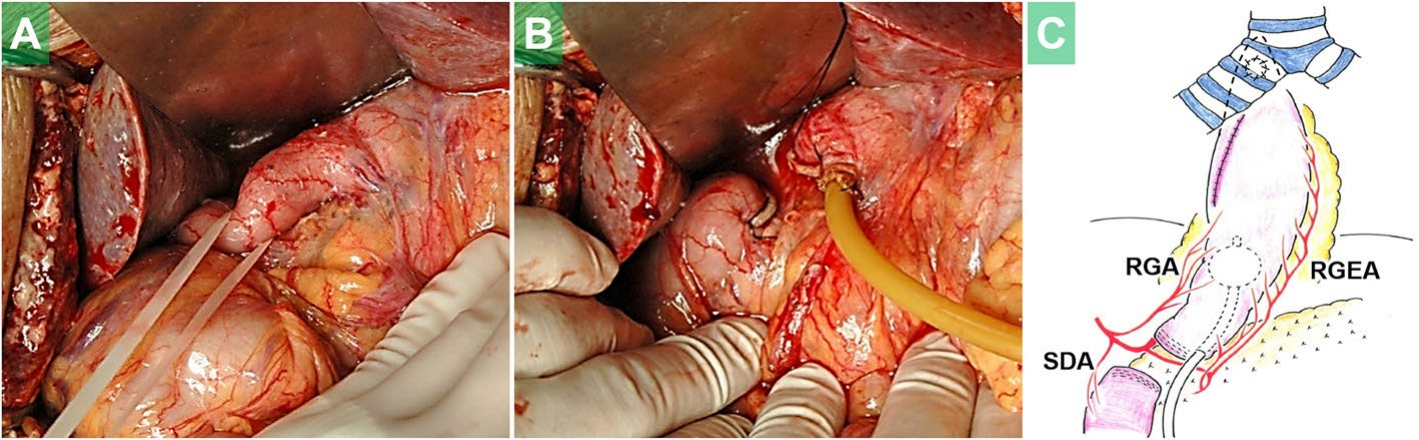
The duodenum was exposed with minimal devascularization and circumferentially taped (**A**). The duodenum was divided with a linear stapler. Finally, a Foley catheter was inserted into the gastric conduit for decompression gastrostomy (**B**). A schematic presentation (**C**). RGA: right gastric artery; RGEA: right gastroepiploic artery; SDA: supraduodenal artery

This operation transiently restored the patient’s respiratory function. Nine days later, however, marked bile reflux into the trachea and pleural space was detected, in association with collapse of the remaining stomach and re-fistulization to the airway. In an effort to block the gastroduodenal reflux and respiratory contamination, emergency DD surgery was performed (operative time 71 min, blood loss unmeasurable) (Fig. 2).

After the DD procedure, his pulmonary condition gradually improved without digestive juice reflux into the airway. The patient was transferred to the general ward 27 days after DD and was discharged on the 77th post-DD day with tube feeding via jejunostomy enteral access. To date (14 months after DD), the patient’s general status remains good and an elective reconstructive operation using the intestinal flap is planned.

### Case 2

A 77-year-old man, with a history of diabetes mellitus, underwent robot-assisted trans-mediastinal esophagectomy with posterior mediastinal gastric tube replacement for EC (squamous cell carcinoma, cT3N1M0, post neoadjuvant chemotherapy). Cervical anastomotic leakage was radiographically identified on the 8th POD. On the 23rd POD, a markedly increased sputum amount with elevated oxygen demand became apparent. Subsequent EGD and bronchoscopy clearly showed TGF formation.

**Fig. 3:**
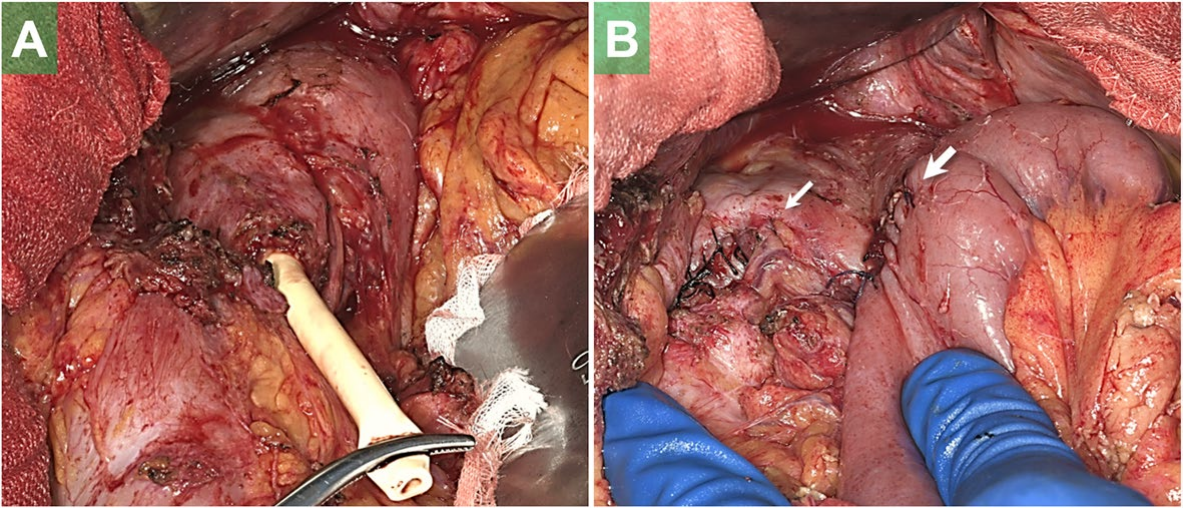
The stump of the duodenum (gastric conduit side) with a gastrostomy tube was identified (**A**). The tube was removed and the orifice was closed with a linear stapler. The stump was reinforced with seromuscular sutures (*thin arrow*). Gastrojejunostomy was created on the anterior wall of the gastric conduit (*thick arrow*) (**B**)

The following day (24th POD), DD surgery, along with pectoralis major muscle implantation sealing the fistula and tube jejunostomy, was completed with a 438-min procedure and 1220-mL blood loss. Ventilator weaning was achieved 25 days after the DD procedure and the patient’s respiratory condition remained stable thereafter. Seven months after DD, when complete TGF closure was confirmed based on radiographic and endoscopic examinations, elective reconstruction surgery using R-Y gastrojejunostomy was performed (operative time 294 min, blood loss 280 mL) (Fig. 3). The postoperative course was uneventful. Oral intake was resumed on the 25th POD and steadily advanced since then.

## Discussion

Various etiological factors, including gastric/tracheobronchial ischemia and anastomotic leakage, play a role in TGF as in our cases and it can be present as not only early but also late post-esophagectomy complications [2–5, 16–18]. However, this challenging comorbidity is not very common and the number of relevant studies thus remains limited. The incidence of TGF development in esophagectomized patients reportedly ranges from 0.3 to 1.5% [3, 7, 12, 18], which is consistent with the rate in our institution (0.7%). As a consequence of this relatively low incidence, no consensus has yet been reached, nor have any firm treatment principles been established, on the optimal management of TGF.

Conventional approaches to TGF are classified into surgical and non-surgical procedures. Surgical management strategies consist of primary closure of the fistula, pedicled muscle flap interposition and/or occasionally esophagogastric resection [3, 4, 7, 11, 12]. Non-surgical therapy is comprised of trachea-brachial stent placement or conservative treatment [4–6, 9–12]. Surgical intervention has been advocated as being preferrable for managing TGF because of the clearer effect and lower fistula recurrence rate [10, 17]. Airway stenting is certainly subject to relapse owing to poor fixation, cover rupture and migration, such that surgical repair appears to be more reliable and to achieve better survival rates [11]. Nevertheless, even with respect to surgical interventions, survival outcomes reportedly remain very poor (9-month survival rate, 38%) [11] and an innovative strategy which could be added to existing approaches is thus highly anticipated. Our clinical experiences raise the possibility that DD might be a key strategy for managing patients in whom stent insertion and/or surgical repair has failed.

According to our literature search, DD has not previously been described as a treatment option for TGF. As indicated in our series, the DD procedure is not radical but, rather, is a temporary management approach against bile reflux potentially worsening TGF status [3]. In fact, DD requires metachronous re-reconstruction to reestablish oral food intake. Therefore, DD is not essential unless conventional treatments fail, and may serve as a means of crisis avoidance. The therapeutic advantages are (i) stopping gastroduodenal reflux, (ii) reducing the inflammatory environment around the TGF and (iii) securing time for TGF resolution while providing enteral nutrition. Such complete prevention of bile reflux from the duodenum is unfeasible with nasogastric tube drainage alone. With regards to our series, DD was feasible even when the physiological conditions of the patients had deteriorated markedly. Reversal of and recovery from severe pulmonary contamination were achieved in both cases. However, oral intake was restored in only one of our cases, such that post-DD dietary resumption cannot be fully guaranteed. As yet, the feasibility of a second reconstruction after DD surgery remains uncertain and further accumulation of experience with this procedure in clinical practice is necessary before a new treatment algorithm can be established.

In summary, we retrospectively reviewed our experiences with DD surgery for managing post-esophagectomy TGF. DD procedures were feasible even in patients whose conditions had deteriorated markedly after esophagectomy, and appeared to exert beneficial effects in terms of eliminating gastroduodenal reflux and promoting recovery of respiratory function. DD might be applicable as an evacuation treatment option when respiratory inflammation persists with conventional therapy alone.

## Data Availability

Not applicable.

## Abbreviations

CT: Computed tomography
DD: Duodenal diversion
EC: Esophageal cancer
EGD: Esophagogastroduodenoscopy
POD: Postoperative day
TGF: Tracheobroncho-gastric fistula
